# Neuroradiological Changes Following Single or Repetitive Mild TBI

**DOI:** 10.3389/fnsys.2019.00034

**Published:** 2019-08-02

**Authors:** Praveen Kulkarni, Thomas R. Morrison, Xuezhu Cai, Sade Iriah, Neal Simon, Julia Sabrick, Lucas Neuroth, Craig F. Ferris

**Affiliations:** ^1^Center for Translational NeuroImaging, Northeastern University, Boston, MA, United States; ^2^Azevan Pharmaceuticals, Bethlehem, PA, United States; ^3^Department of Biological Sciences, College of Arts and Sciences, Lehigh University, Bethlehem, PA, United States

**Keywords:** Parkinson’s disease, dopamine, dementia, hyperconnectivity, microglia activation, cerebellum, suprachiasmatic nucleus (SCN), olfactory system diseases

## Abstract

**Objectives:**

To test the hypothesis that there are differences in neuroradiological measures between single and repeated mild traumatic brain injury using multimodal MRI.

**Methods:**

A closed-head momentum exchange model was used to produce one or three mild head injuries in young adult male rats compared to non-injured, age and weight-matched controls. Six–seven weeks post-injury, rats were studied for deficits in cognitive and motor function. Seven–eight weeks post-injury changes in brain anatomy and function were evaluated through analysis of high resolution T2 weighted images, resting-state BOLD functional connectivity, and diffusion weighted imaging with quantitative anisotropy.

**Results:**

Head injuries occurred without skull fracture or signs of intracranial bleeding or contusion. There were no significant differences in cognitive or motors behaviors between experimental groups. With a single mild hit, the affected areas were limited to the caudate/putamen and central amygdala. Rats hit three times showed altered diffusivity in white matter tracts, basal ganglia, central amygdala, brainstem, and cerebellum. Comparing three hits to one hit showed a similar pattern of change underscoring a dose effect of repeated head injury on the brainstem and cerebellum. Disruption of functional connectivity was pronounced with three mild hits. The midbrain dopamine system, hippocampus, and brainstem/cerebellum showed hypoconnectivity. Interestingly, rats exposed to one hit showed *enhanced* functional connectivity (or hyperconnectivity) across brain sites, particularly between the olfactory system and the cerebellum.

**Interpretation:**

Neuroradiological evidence of altered brain structure and function, particularly in striatal and midbrain dopaminergic areas, persists long after mild repetitive head injury. These changes may serve as biomarkers of neurodegeneration and risk for dementia later in life.

## Introduction

Traumatic brain injuries (TBIs) are responsible for over 2.8 million emergency room visits and 50,000 deaths in the United States each year (Taylor et al., 2017). Mild TBI is characterized as a negligible loss of consciousness with minimal neuropathology (Ruff et al., 2009; Menon et al., 2010) and is estimated to account for 70–90% of all TBI cases (Gardner and Yaffe, 2015; Astafiev et al., 2016). Mild TBI following a single incident is difficult to detect, most cognitive and behavioral deficits usually resolve within weeks of the head injury, and few cases result in extended recovery time periods (Lovell et al., 2003; McCrea et al., 2003; Iverson, 2005; Losoi et al., 2016). However, a more pernicious, long-lasting condition may arise with repeated incidents of mild TBI (rmTBI) (Guskiewicz et al., 2003). Repeated mild TBI is associated with more severe and protracted cognitive, motor, and behavioral complications that may last for months and even years (De Beaumont et al., 2009, 2012; Omalu et al., 2010). Even after the remission of symptoms, there is accumulating evidence of persistent brain injuries (Nakamura et al., 2009; Mayer et al., 2011; Palacios et al., 2017; Rajesh et al., 2017; Vergara et al., 2017) that carry an increased risk of dementia, including Alzheimer’s disease, chronic traumatic encephalopathy, and Parkinson’s disease later in life (Plassman et al., 2000; McKee et al., 2009; Gavett et al., 2011; Konrad et al., 2011; Sivanandam and Thakur, 2012; Jafari et al., 2013; Faden and Loane, 2015; Gardner and Yaffe, 2015; Jenkins et al., 2018).

The objective of this study was to use a momentum exchange model of head injury in rat to characterize neuroradiological differences between single and repeated mild TBI. To this end, we used diffusion weighted imaging (DWI) with indices of anisotropy registered to a 3D MRI rat atlas and computational analysis to identify putative changes in gray matter microarchitecture across 173 brain areas in control and experimental rats hit one or three times. In addition, resting-state BOLD functional connectivity (rsFC) was employed to evaluate alterations in global functional neural circuitry. These MRI protocols were selected due to their clinical use in diagnosing and following the progression of rmTBI after remission of symptoms (Nakamura et al., 2009; Mayer et al., 2011; Palacios et al., 2017; Rajesh et al., 2017; Vergara et al., 2017) as well as their utility in identifying biomarkers of neurodegenerative disease (Wu et al., 2009; Hacker et al., 2012; Koch et al., 2012; Teipel et al., 2013; Zhang et al., 2015). We adapted the momentum exchange model developed by the National Football League to study player concussions and designed for preclinical studies by Viano et al. (2009) to scale to humans. The velocity of head movement and energy transfer was calculated and scaled to mimic a mild concussive injury in humans. This injury was defined by the absence of skull fractures, prolonged loss of consciousness, or signs of intracranial bleeding, which are seen in TBIs classified as moderate or severe (Hardman and Manoukian, 2002).

## Materials and Methods

### Animals

Adult, male Sprague Dawley rats (300–400 g) were purchased from Charles River Laboratories (Wilmington, MA, United States). Animals were housed in Plexiglas cages (two per cage) and maintained in ambient temperature (22–24°C) on a 12:12 light:dark cycle (lights on at 07:00 a.m.). Food and water were provided *ad libitum*. All methods and procedures described were approved by the Northeastern University Institutional Animal Care and Use Committee (IACUC). The Northeastern facility is AAALAC accredited with OLAW Assurance and is registered with the USDA. All housing, care, and use followed the Guide for the Care and Use of Laboratory Animals (8th Addition) and the Animal Welfare Act.

### Momentum Exchange Model

Working with engineers at Animals Imaging Research, LLC (Holden, MA, United States), we replicated the pneumatic pressure drive, 50 g compactor (see Supplementary Figure 1) described by Viano et al. (2009) and reliably produced the 7.4, 9.3, and 11.2 m/s impact velocities described for mild, medium, and severe rat head injury, respectively. This same model was further refined and used to test the behavioral effects of mild TBI controlling for the axis of injury, rotational force, and head acceleration in different directions(Mychasiuk et al., 2016). Our impact created linear acceleration with some rotation. The data reported here all came from the 7.4 m/s impact velocities as determined using high-speed video recordings. The impact piston was directed to the top of the skull, midline, in the approximate area of Bregma. All control and TBI rats were anesthetized with 2% isoflurane. Rats were awake and ambulatory within 5–7 min after anesthesia and concussion. This impact regimen produced no signs of contusion (see Supplementary Figure 2). Rats were observed twice daily, in the morning and early evening, for the first week after TBI and weekly thereafter. Body weights were taken two to three times/week for the first week and then weekly. Buprenorphine treatment was available for pain and distress, but it was deemed unnecessary based upon behavioral observations and response to handling. There were no unplanned mortalities over the course of the study.

The impact regimen was based on a rich body of data detailing the effects of acute and rmTBI in various rodent models (Shultz et al., 2012; Xiong et al., 2013; Aungst et al., 2014; Fidan et al., 2016). Studies were scheduled one (*n* = 13) or three (*n* = 9) concussive head impacts under 2% isoflurane anesthesia, with a 48-h interval between each impact. Control rats (*n* = 9) were exposed to isoflurane anesthesia three times with 48 h intervals to control for the effects of anesthesia. Rats were not tested for neurological deficits after head injury; instead, they were returned to their home cage after their final TBI and left undisturbed for 6 weeks. Between 6 and 7 weeks after head injury, all animals were tested for cognitive and motor behavior. Between 7 and 8 weeks post injury all animals were imaged. Rats were euthanized with a combination of carbon dioxide asphyxiation until the cessation of respiration followed by thoracotomy.

### Neuroimaging

Imaging sessions were conducted using a Bruker Biospec 7.0T/20-cm USR horizontal magnet (Bruker, Billerica, MA, United States) and a 20-G/cm magnetic field gradient insert (ID = 12 cm) capable of a 120-μs rise time. Radio frequency signals were sent and received with a quadrature volume coil built into the animal restrainer (Animal Imaging Research, Holden, MA, United States). The design of the restraining system included a padded head support obviating the need for ear bars helping to reduce animal discomfort while minimizing motion artifact. All rats were imaged under 1–2% isoflurane while keeping a respiratory rate of 40–50/min. At the beginning of each imaging session, a high-resolution anatomical data set was collected using the RARE pulse sequence with following parameters, 35 slice of 0.7 mm thickness; field of view (FOV) 3 cm; 256 × 256; repetition time (TR) 3900 ms; effective echo time (TE) 48 ms; NEX 3; 6 min 14 s acquisition time.

### Diffusion Weighted Imaging – Quantitative Anisotropy

Diffusion weighted imaging was acquired with a spin-echo echo-planar-imaging (EPI) pulse sequence having the following parameters: TR/TE = 500/20 ms, eight EPI segments, and 10 non-collinear gradient directions with a single *B*-value shell at 1000 s/mm^2^ and one image with a *B-*value of 0 s/mm^2^ (referred to as *B*_0_). Geometrical parameters were: 48 coronal slices, each 0.313 mm thick (brain volume) and with in-plane resolution of 0.313 × 0.313 mm^2^ (matrix size 96 × 96; FOV 30 mm^2^). The imaging protocol was repeated two times for signal averaging. Each DWI acquisition took 35 min and the entire MRI protocol lasted ca. 70 min. Image analysis included DWI analysis of the DW-3D-EPI images to produce the maps of fractional anisotropy (FA) using a 3D MRI Rat Brain Atlas©(Ekam Solutions LLC, Boston, MA, United States). DWI analysis was completed with MATLAB and MedINRIA (1.9.0^1^) software. Because sporadic excessive breathing during DWI acquisition can lead to significant image motion artifacts that are apparent only in the slices sampled when motion occurred, each image (for each slice and each gradient direction) was screened, prior to DWI analysis. If found, acquisition points with motion artifacts were eliminated from analyses.

For statistical comparisons between rats, each brain volume was registered to the 3D rat atlas allowing voxel- and region-based statistics. All image transformations and statistical analyses were carried out using the in-house MIVA software^2^. For each rat, the *B*_0_ image was co-registered with the *B*_0_ template (using a six-parameter rigid-body transformation). The co-registration parameters were then applied on the DWI indexed maps for the different indices of anisotropy. Normalization was performed on the maps since they provided the most detailed visualization of brain structures and allowed for more accurate normalization. The normalization parameters were then applied to all DWI indexed maps that were then smoothed with a 0.3-mm Gaussian kernel. To ensure that FA and RD values were not affected significantly by the pre-processing steps, the “nearest neighbor” option was used following registration and normalization.

Statistical differences in measures of DWI between experimental groups were determined using a nonparametric Mann–Whitney *U*-test (alpha set at 5%). The formula below was used to account for false discovery from multiple comparisons.

$$P\left(i\right)\leq \frac{i}{V}\frac{q}{c\left(V\right)},$$

where *P*(*i*) is the *p-*value based on the *t-*test analysis. Each of 171 regions of interest (ROIs) (*i*) within the brain containing (*V*) ROIs was ranked in order of its probability value (Table 1). The false-positive filter value *q* was set to 0.2 and the predetermined *c*(*V*) was set to unity (Benjamini and Hochberg, 1995). The corrected probability is noted on each table.

**TABLE 1:** Measures of motor behavior.

**Apparatus and parameter**	**Control**	**1-Hit**	**3-Hit**	**Statistics**
**Balance beam**				
Total foot faults	2.0 ± 0.7	2.2 ± 0.6	2.8 ± 0.9	*F* = 0.31, *p* = 0.74^a^
**Faults per segment:**				
Wide	0.3 ± 0.1	0.5 ± 0.2	0.5 ± 0.2	–
Middle	0.2 ± 0.1	0.1 ± 0.1	0.3 ± 0.2	*p* < 0.01^b^
Thin	0.5 ± 0.2	0.6 ± 0.1	0.6 ± 0.1	*p <* 0.02^b^
Goal box latency (s)	12.7 ± 2.2	9.9 ± 1.2	14.7 ± 3.7	*F* = 0.99, *p* = 0.38^a^
**Rota-rod**				
Fall latency (s)	107.8 ± 9.7	83.2 ± 9.6	100.5 ± 19.8	*F* = 1.05, *p* = 0.36^a^

*Data represent mean ± SEM. For balance beam testing, there were no significant differences between groups for goal box latency [*F*(2,27) = 0.99, *p* > 0.1] or total foot faults [*F*(2,27) = 0.30, *p* > 0.1]. Similarly there was no difference between groups for fall latency on the rota-rod task [*F*(2,27) = 1.05, *p* > 0.1]. Analysis of balance beam performance, in terms of the widths of the three specific beam segments (i.e., Wide, Middle, and Thin), resulted in a significant main effect of segment width [*F*(2,52) = 11.7, *p* < 0.0001] with all groups showing a higher number of foot faults on the thinnest and middle portion of the beam compared to the widest portion of the beam (*p* < 0.05 for both). ^*a*^Non-significant; ^*b*^significant.*

### Resting-State Functional Connectivity

Scans were collected using a spin-echo triple-shot EPI sequence [imaging parameters: matrix size = 96 × 96 × 20 (H × W × D), TR/TE = 1000/15 ms, voxel size = 0.312 × 0.312 × 1.2 mm, slice thickness = 1.2 mm, with 200 repetitions, time of acquisition 10 min]. There are numerous studies detailing the benefits of multi-shot EPI in BOLD imaging (Menon et al., 1997; Hoogenraad et al., 2000; Poser and Norris, 2009; Swisher et al., 2012; Kang et al., 2015). We avoided using single shot EPI because of its sever geometrical distortion at high field strengths (≥7T) and loss of effective spatial resolution as the readout period increases (Farzaneh et al., 1990; Jesmanowicz et al., 1998; Hoogenraad et al., 2000). There is also the possibility of signal loss in single shot EPI due to accumulated magnetic susceptibility or field inhomogeneity (Kang et al., 2015).

Preprocessing in this study was accomplished by combining Analysis of Functional NeuroImages (AFNI_17.1.12^3^), FMRIB Software library (FSL, v5.0.9^4^), Deformable Registration via Attribute Matching and Mutual-Saliency Weighting (DRAMMS 1.4.1^5^), and MATLAB (Mathworks, Natick, MA, United States). Brain tissue masks for resting-state functional images were manually drawn using 3DSlicer^6^ and applied for skull-stripping. Motion outliers (i.e., data corrupted by extensive motion) were detected in the dataset and the corresponding time points were recorded so that they could be regressed out in a later step. Functional data were assessed for the presence of motion spikes. Any large motion spikes were identified and removed from the time-course signals. This filtering step was followed by slice timing correction from interleaved slice acquisition order. Head motion correction (six motion parameters) was carried out using the first volume as a reference image. Normalization was completed by registering functional data to the 3D MRI Rat Brain Atlas©using affine registration through DRAMMS. The MRI rat atlas containing 173 annotated brain regions was used for segmentation. Data are reported in 166 brain areas, as five regions in the brain atlas were excluded from analysis due to the large size of three brains. These brains fell slightly outside our imaging FOV and thus we did not get any signal from the extreme caudal tip of the cerebellum. Whole brains that contain all ROIs are needed for analyses so rather than excluding the animals, we removed the brain sites across all animals. After quality assurance, band-pass filtering (0.01–0.1 Hz) was preformed to reduce low-frequency drift effects and high-frequency physiological noise for each subject. The resulting images were further detrended and spatially smoothed (full width at half maximum = 0.8mm). Finally, regressors comprised of motion outliers, the six motion parameters, the mean white matter, and cerebrospinal fluid time series were fed into general linear models for nuisance regression to remove unwanted effects.

The region-to-region functional connectivity method was performed in this study to measure the correlations in spontaneous BOLD fluctuations. A network is comprised of nodes and edges; nodes being the brain ROI and edges being the connections between regions. Data are reported in 166 brain areas, as five regions in the 3D MRI Rat Brain Atlas were excluded from analysis due to the large size of three brains that fell slightly outside then FOV excluding signal from the most caudal tip of the cerebellum. Voxel time series data were averaged in each node based on the residual images using the nuisance regression procedure. Pearson’s correlation coefficients across all pairs of nodes (14,535 pairs) were computed for each subject among all three groups to assess the interregional temporal correlations. The *r*-values (ranging from −1 to 1) were *z*-transformed using the Fisher’s *Z* transform to improve normality. 166 × 166 symmetric connectivity matrices were constructed with each entry representing the strength of edge. Group-level analysis was performed to look at the functional connectivity in the experimental groups. The resulting *Z*-score matrices from one-group *t*-tests were clustered using the K-nearest neighbors clustering method to identify how nodes cluster together and form resting-state networks. A *Z*-score threshold of |*Z*| = 2.3 was applied to remove spurious or weak node connections for visualization purposes.

### Behavioral Testing

The novel object recognition (NOR) task was used to assess episodic learning and memory (Bevins and Besheer, 2006; Antunes and Biala, 2012). The apparatus consisted of a black cube-shaped Plexiglass box (L: 60.9, W: 69.2, H: 70.5 cm) with no lid, indirectly illuminated with two 40 W incandescent bulbs. Animals were placed in the empty box (15 min) for acclimation on day 1. On day 2, for the familiar phase (5 min), animals were placed in the box with two identical objects arranged in diagonal corners, 5 cm from each wall. After a 90 min rest period in their home cage, animals were placed back in the box for the novel phase (3 min) with one of the familiar objects and a novel object.

The Barnes Maze was used to assess spatial learning and memory (Barnes, 1979; Fox et al., 1998; Harrison et al., 2009). The maze consists of a circular platform (121 cm in diameter, elevated 40 cm), with 18 escape holes along the perimeter at 30 cm intervals. A black, removable enclosed Plexiglas goal box was positioned under a single escape hole on the underside of the maze (L:40.0 × W:12.7 × H:7.6 cm) in the same position relative to the testing room across all trials. Between trials, the maze was rotated 45 degrees and the goal box shifted accordingly for cardinal consistency. Animals were placed inside the goal box for 1 min and then under an enclosed container at the center of the circular platform for 30 s, that was then lifted to start the trial. If animals did not find the goal box within the test period (4 min), they were gently nudged into the box and allowed to stay for 1 min, and then placed back in their home cages between trials (three trials/day for 4 days). For both the NOR and the Barnes maze, all trials were video recorded and analyzed using manual methods by experimenters blind to treatment condition and verified with automated scoring using ANY-maze^®^ software (Stoelting, Wood Dale, IL, United States).

A tapered balance beam (Dragonfly Inc., Ridgeley, WV, United States) and rota-rod were used to measure motor behavior (Williams et al., 2005; Sackheim et al., 2017). The balance beam (L: 150 cm, W: 5.5 cm tapering down to 1.5 cm, elevated 120 cm) was equally divided into three sections (L:47 cm each; “wide,” “middle,” “thin” sections) that were lined with touch-sensitive sensor ledges (width: 2 cm) that ran the length of the beam and were arranged on each side, 4 cm below the surface of the beam to count paw slips (or *foot faults*). At the start of the maze (“wide” section) was a wooden start platform, and at the end of the beam (immediately following the “thin” section) was a black enclosed Plexiglas goal box. After 2 days of training (three trials per day), animals were tested (three trials/day for 2 days). Prior to each trial, animals were placed inside the goal box for 1 min. Animals were then placed on a start platform and timed for traversing into the goal box, where they remained for 1 min, and were then placed back in their home cage until the next trial (30 min intertrial interval).

Following 2 days of training (three trials/day), animals were tested over 2 days (three trials/day) using the rota-rod by placing them on a rotating cylinder (diameter: 4 cm) that rotated at an increasing frequency starting at 1 rpm and increasing linearly at a 0.1 v/t2 acceleration rate for a total of 210 s ending at a max frequency of 50 rpm. Latency to fall off the rod was recorded for each animal and averaged across trials and days. For all behavioral measures, GraphPad Prism version 6.0 (GraphPad Software, La Jolla, CA, United States) was used for statistical analyses. One-sample *t*-tests assessed differences from chance levels (i.e., =50%) of exploration in the NOR task, for each experimental group individually. Comparisons among groups were conducted using one-way analysis of variance (ANOVA) or mixed ANOVAs followed by Fisher’s protected least significant difference *post hoc* test.

## Results

### Cognitive and Motor Behavior

**FIGURE 1:**
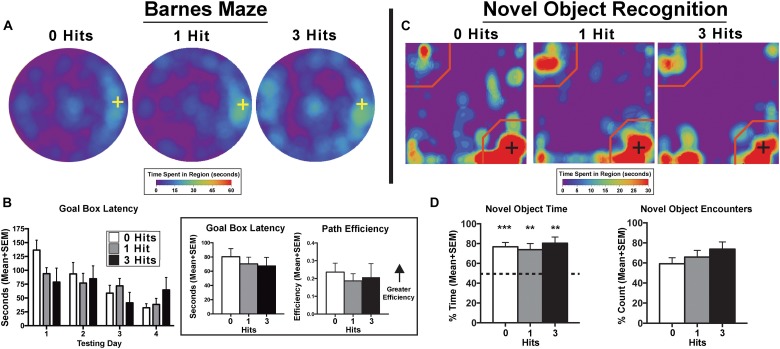
Cognition testing. **(A)** Barnes maze heat maps for the last day of testing. Qualitatively, on the last day of testing, animals exposed to three hits spent more time on the maze searching for the goal box, however, this effect failed to reach quantitative significance. **(B)** Search patterns for the Barnes maze goal box resulted in no significant differences between groups across days for all maze parameters including goal box latency and path efficiency. There was a main effect of path efficiency on the Barnes maze with all experimental groups showing relatively steady increases across days [*F*(3,81) = 3.29, *p* < 0.05] (Inset). When averaged over days, there were no significant differences between groups for goal box latency [*F*(2,27) = 0.20, *p* = 0.82] or path efficiency [*F*(2,27) = 0.22, *p* = 0.80]. **(C)** Novel object recognition (NOR) – novel phase heat maps (averaged across days). Qualitative data show an equal pattern of exploration across groups with the greatest amount of time spent in close proximity to the novel object indicated by the presence of red on the lower right corner of the maps. **(D)** Quantitative data reflect qualitative patterns and show that all three treatment groups spent a greater than chance amount of time (i.e., >50%) with the novel object. Conversely, there were no differences between hit groups for the NOR index [*F*(2,27) = 0.34, *p* > 0.1], total time exploring the novel object [*F*(2,27) = 0.57, *p* > 0.1], or the percentage of novel object encounters during the novel phase [*F*(2,27) = 1.16, *p* > 0.1]. Crosses on heat maps indicate goal box location and novel object location on the Barnes maze and NOR, respectively. ^∗∗^*p <* 0.05, ^∗∗∗^*p <* 0.01 for single sample *t*-test.

**FIGURE 2:**
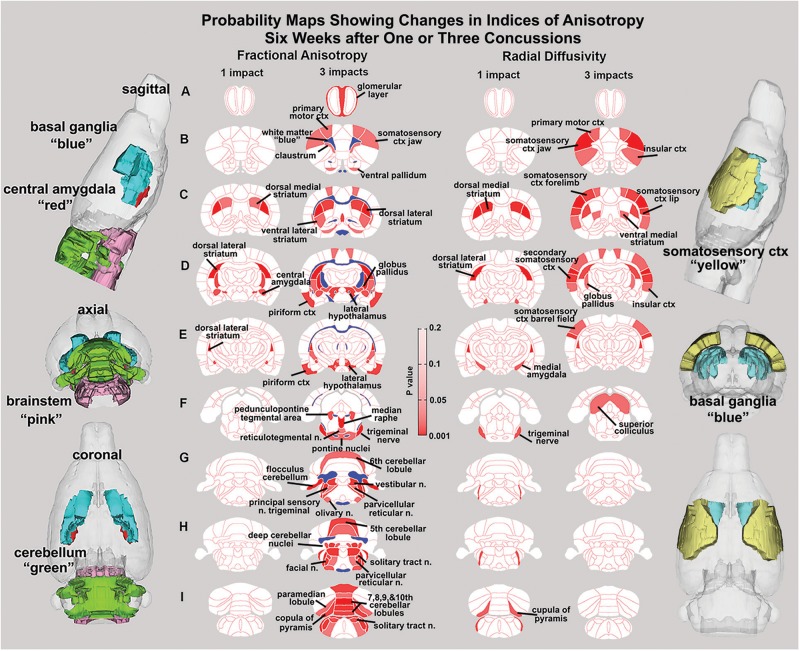
Diffusion weighted imaging. Shown are 2D probability maps with quantitative anisotropy highlighting the brain areas (pink/red) that are significantly different in FA between one (*n* = 13) and three (*n* = 9). Most of these areas are associated with the basal ganglia, cerebellum, and brainstem. The 3D representations of these areas are presented in different orthogonal directions to the left. The forebrain areas shown in sections **B–D** are near the impact site and include the underlying ctx and striatum (caudate/putamen), while the hindbrain areas (sections **F–I**) include various components of the pons and medulla oblongata that are associated with arousal (raphe, parvicellular reticular, pedunculopontine tegmental, reticulotegmental areas), sensory integration (principle sensory n, facial n, vestibular n.), and autonomic regulation (solitary tract n.). The pontine and olivary nuclei have efferent connections to the cerebellum as do the many sensory nuclei in medulla. The posterior cerebellum comprising the vermis (5th–10th cerebellar lobules), flocculus, paramedian lobules, cupola of the pyramis, and deep cerebellar n. were all affected with three hits.

**FIGURE 3:**
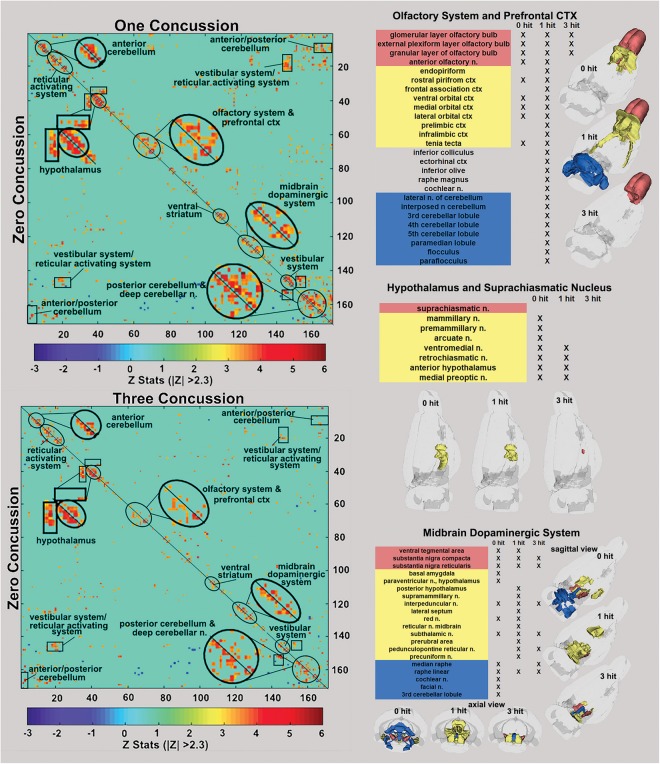
Functional connectivity correlation matrices. Shown are correlation matrices of 166 rat brain areas for rsFC comparing controls to one concussion (top left) and three concussions (bottom left). Each dark red pixel for control rats represents 1 of 166 brain areas that is significantly correlated with other brain areas. The brain areas with significant correlations appear as clusters because they are contiguous in their neuroanatomy and function. The diagonal line separates the control and one concussed groups. The pixels for one concussion are a mirror image of those pixels (i.e., brain areas for controls). Interesting, rats with a single concussion (*n* = 13) showed greater rsFC within the anterior and posterior cerebellum and deep cerebellar n., olfactory system, and prefrontal ctx when compared to no hit controls (*n* = 9).

Across days, there was a significant main effect of testing day on goal box latency in the Barnes maze test [*F*(3,81) = 9.3, *p* < 0.0001], with no significant difference between groups [*F*(2,27) = 0.38, *p* > 0.1, Figure 1]. All groups had significantly shorter latencies to enter the goal box on testing day 3 (*p* < 0.0001), and 4 (*p* < 0.0001) compared to day 1. In addition, all groups showed shorter latencies on the last day of testing compared to the second day of testing (*p* < 0.01). In the NOR, single-sample *t*-tests showed that control, one, and three hit animals [*t*(11) = 6.84, *p* < 0.0001; *t*(9) = 3.86, *p* < 0.01; and *t*(7) = 4.9, *p* < 0.001, respectively] all had a significantly greater preference for the novel object that was beyond chance (>50%) during the novel phase (Figure 1). The DWI and rsFC data showed no evidence of alterations in the hippocampal complex as shown in Figures 2, 3. Table 1 summarizes the results of locomotor testing and shows no differences between the groups.

### Diffusion Weighted Imaging and Quantitative Anisotropy

Measures of anisotropy at 7–8 weeks post injury were registered to the 3D MRI Rat Atlas with 173 segmented brain areas to identify possible changes in gray matter microarchitecture (Kulkarni et al., 2015). The data for FA are shown in Figure 2. These probability heat maps show statistical differences between the one and three hit groups compared to controls. The right column of activation maps shows three hits compared to one hit. The effects on FA from a single hit were limited to the dorsal striatum and central amygdala. However, rats exposed to three hits showed significant FA changes in the olfactory system, basal ganglia, central amygdala, cerebellum, and deep cerebellar nuclei. Many of the same differences are noted when comparing three hits to one hit, evidence of a dose effect with repeated mild head injuries (see Supplementary Tables 1–3).

### Resting-State Functional Connectivity

The delineated areas in the two correlation matrices in Figure 3 show that a single hit favors an increase in rsFC, while repeated hits show reduced rsFC. For example, the posterior cerebellum of the one hit group shows a much larger cluster than both the three hit and control animals. Indeed, this area has grown to include the paramedian lobule, crus 1 and 2, cerebellar lobules 7, 8, 9, and 10 plus the deep cerebellar nuclei. The rsFC between brain regions for the three experimental groups are shown in the right-hand panels of Figure 3 for the olfactory system/prefrontal cortex, suprachiasmatic n. (SCN) of the hypothalamus, and the midbrain dopaminergic system. The areas in red comprise the key nodes for each panel. For instance, the olfactory system is made up of the three layers of the olfactory bulb and the anterior olfactory nucleus. In control rats, these combined areas have significant functional connections to the marked areas of the adjacent prefrontal ctx (e.g., rostral piriform, ventral, medial and lateral orbital cortex, and the tenia tecta, highlighted in yellow). The 3D organization of these brain areas and the others is shown in the glass brains. Rats hit once showed increase functional connectivity 7 weeks post injury that includes the anterior cerebellum (three to five lobules) and deep cerebellar nuclei (lateral and interposed). In contrast, rats exposed to three hits have reduced connectivity that is limited only to the olfactory bulb. The SCN, the key node in the brain controlling circadian rhythms and sleep/wake cycles, has functional connections with adjacent areas of the hypothalamus in control rats that are reduced with one hit and eliminated with three hits.

The ventral tegmental area (VTA) as well as the substantia nigra compacta (SNc) and reticularis (SNr) make up the core nodes of the midbrain dopaminergic system. From these regions, control animals have diffuse connectivity to areas in the amygdala, hypothalamus, thalamus, medulla oblongata, and cerebellum. Following a single mild hit, the functional connectivity primarily coalesces around the thalamus. Animals exposed to repeated TBI showed reduced connectivity compared to the other groups, and had no connectivity between the SN and the VTA.

**FIGURE 4:**
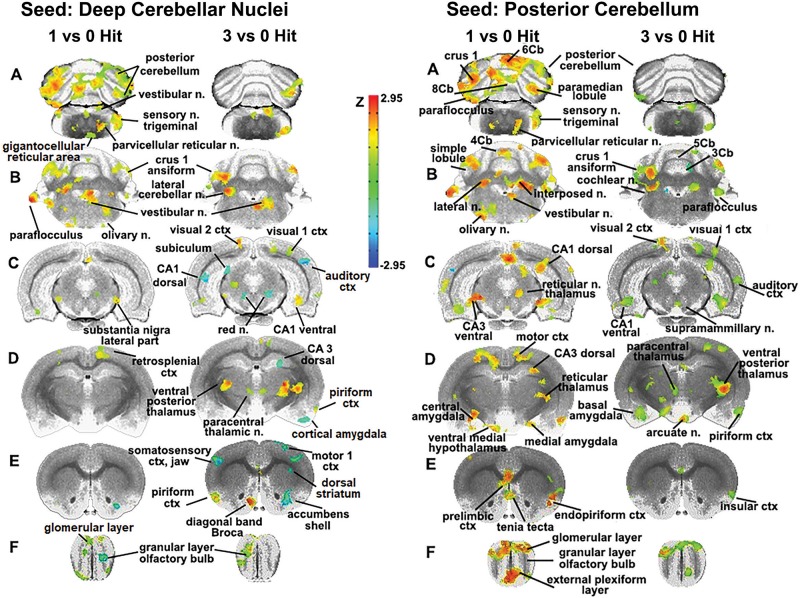
Seeding the cerebellum. The deep cerebellar nuclei (lateral, fastigial, and interposed) and collective areas comprising the posterior cerebellum served as seeds point to focus on the connectivity of the brain to the cerebellum following one and three concussions (hits) as compared to 0 hit sham controls. Areas denoted in red/yellow are significantly greater than control while blue are significantly less than control. Sections **A** and **B** show increased connectivity between the deep cerebellar nuclei and the posterior cerebellum (lobules 5–9), crus 1 of the ansiform lobule, and paraflocculus in one hit rats. Additionally, the primary sensory n. of the trigeminal nerve, vestibular n., parvicellular reticular n., and the olivary n. of the underlying medulla oblongata are all part of the enhanced functional connectivity that is present in one hit rats compared to control animals. These same cerebellar/brain stem connections were reduced in the three hit rats (controls < three hit < one hit, sections **A** and **B**). At the level of the pons (section **C**) there is clear bilateral connectivity to the lateral part of the SN in one hit rats and, to a lesser degree, a unilateral connection in the three hit rats. Three hit rats show a reduced connectivity (blue) in the red n., dorsal CA1, and dorsal subiculum of the hippocampus (three hit < control) but an increased connectivity in the ventral CA1, visual 1 and 2 cortices (control < three hit). At the level of the thalamus (section **D**) there is only one area that differs between one hit and control animals, the retrosplenial ctx. In contrast, the three hit rats show enhanced bilateral connectivity in the ventral posterior thalamus and paracentral thalamic n., and reduced connectivity in dorsal CA3 hippocampus and piriform ctx. At the level of the striatum (section **E**) there is reduced connectivity in one and three hit rats in the accumbens shell (one hit ≤ three hit < control). Three hit rats show a bilateral reduction in the primary somatosensory cortex representing the jaw, a unilateral reduction in connectivity to the motor ctx, and enhanced connectivity in the piriform ctx and diagonal band of Broca.

The sensitivity of the cerebellum and its efferent connections to the brain through the deep cerebellar nuclei was examined further by seeding the combined lateral, fastigial, and interposed nuclei, and mapping areas of connectivity in the one and three hit groups that were significantly different from control (Figure 4). In addition, the posterior cerebellum was also seeded using an aggregate of multiple areas (6–10 lobules, cupola, crus 1 and 2, paramedian, and paraflocculus). The purpose of this seeding strategy was to identify putative afferent connections to the posterior cerebellum given its enhanced functional connectivity following a single concussion. For the one hit group, there was strong connectivity with the olfactory bulb, prelimbic ctx, tenia tecta, and endopiriform ctx (sections E and F). The amygdala (central, medial, and basal, section D), hippocampus (CA3 dorsal and ventral, CA1 dorsal, sections D and C), motor ctx (section D), and medulla oblongata (olivary n., vestibular n. principle sensory n. trigeminal, and parvicellular reticular n., sections A and B) all showed strong connectivity to the posterior cerebellum. These cerebellar connections were fewer and less significant with repeated concussions. The reorganization of functional connectivity in the cerebellum and brainstem shown in Figures 3, 4 compliment the FA data (Figure 2), which showed alterations in water diffusion and putative gray matter microarchitecture across many of the same brain areas. The Excel files with the raw Z scores for all brain areas for zero, one and three hit conditions are provided in Supplementary Tables 4A–C.

## Discussion

The study was designed to evaluate the long-term neuroradiological consequences of one versus three mild repetitive head injuries using the momentum exchange model. The model had the expected effect of delivering repetitive mild head injury without signs of contusion. This model enabled the question – are there any differences between a single mild hit and three mild hits to the head delivered over the course of several days? The imaging data from DWI and rsFC collected 7–8 weeks post injury were very different between a single and three mild hits and suggest a reorganization of gray matter microarchitecture and functional neural circuitry to repetitive head injury.

### One vs. Three TBI

A single, mild TBI caused few changes in indices of anisotropy reflecting minor alterations in central water diffusion. With three concussions, there was evidence of white and gray matter injury, and loss of connectivity between various regions of the brain. This was consistent with several human and animal studies that showed that repetitive injury separated by short intervals poses a greater risk than single insults or multiple head injuries separated by longer intervals (Laurer et al., 2001; Yoshiyama et al., 2005; Meehan et al., 2012; Prins et al., 2013; Silverberg et al., 2013; Bolton and Saatman, 2014; Weil et al., 2014).

The availability and utilization of glucose necessary for brain function following repeated head injury appears to play a critical role in recovery (Selwyn et al., 2016). The change in brain metabolism following injury is triphasic, with an initial period of hyperglycolysis followed by depressed glucose metabolism and finally recovery (Yoshino et al., 1991; Ginsberg et al., 1997; Bergsneider et al., 2000, 2001; Selwyn et al., 2016). The reduction in glucose metabolism may reflect a “dormant” period helping in recovery. In a recent study, Selwyn et al. (2016) looked at repeated head injury in rodents timed to coincided with this “dormant” phase of glucose metabolism and reported greater neurological damage and deficits in motor function compared to injury at other times. Concussions that occur closer together have greater cognitive and behavioral consequences, with deficits that can be present up to year later in preclinical models (Selwyn et al., 2016). In accord with this finding, a repeated imaging study showed that at multiple time points both during and after repeated head strikes, FA and mean diffusivity, as well as axial and radial diffusivity, continue to change across various regions of the brain (Qin et al., 2018).

### Diffusion Weighted Imaging

There were few changes in indices of anisotropy at 7–8 weeks following a single hit. Those that occurred were localized to the central and medial amygdala, and the dorsal/ventral striatum (caudate/putamen). These areas are related to the control of emotion and dopaminergic regulation of motor function, respectively (Lanteaume et al., 2007; Fazio et al., 2011; Smith and Lane, 2015). Rats exposed to three hits showed significant changes in FA within the white matter tracts, olfactory system, basal ganglia, central amygdala, cerebellum, and deep cerebellar nuclei. Comparing three hits to one hit showed a similar pattern of change underscoring a dose effect of repeated head injury on the brainstem and cerebellum. The decrease in FA values in white matter tracts following head injury is well established in the clinical literature (Shenton et al., 2012) and again reported by Wright et al. (2017) in rmTBI using the momentum exchange model and most recently by Fidan et al. (2018) in a closed head cortical impact model. The resulting putative changes to gray matter microarchitecture show a distinct separation between forebrain and hindbrain (see 3D sagittal representation in Figure 2). These results align with numerous reports showing that the cerebellum is particularly vulnerable to mild TBI (Bolton and Saatman, 2014; Ordek et al., 2014; Nathan et al., 2015; Schroeter et al., 2015; Meabon et al., 2016; Manktelow et al., 2017). Furthermore, in a recent study, rsFC data from human mTBI patients identified altered connectivity to the cerebellum as an important biomarker (Vergara et al., 2017). Related to this finding, data from retired military personnel show that decreased metabolic activity in the cerebellum is negatively correlated with the number of blast-related mild TBIs (Selwyn et al., 2016). Given the heterogeneity of TBIs, the consistency of alterations to cerebellar function due to head injury in both humans and across animal models suggests that the cerebellum is an important region for characterizing the progression of head injury.

### Resting-State Functional Connectivity: Midbrain Dopamine System

This study included a global analysis of rsFC of 166 brain regions extending from the rostral-most portion of the olfactory bulb to the caudal brainstem and cerebellum. Animals hit once showed a combination of hyper- and hypoconnectivity across several networks, while rats concussed three times presented with only hypoconnectivity (Figure 3). The altered connectivity of the midbrain dopaminergic system demonstrates injury-dependent hypoconnectivity and reorganization of an extended neural network to a smaller cluster. The VTA and SN make up the core nodes of the midbrain dopaminergic system and have diffuse connectivity to areas in the amygdala, hypothalamus, thalamus, medulla oblongata, and cerebellum in control animals. Following a single concussion, functional connectivity primarily coalesces around the thalamus. This clustering, or “small-world” effect (Bassett and Bullmore, 2009; Nakamura et al., 2009; Roy et al., 2017), shortens the pathway length or aggregate neural connections, reducing the metabolic cost of signal transduction. Repeated concussions show reduced connectivity in these areas compared to one concussion and control groups, as well as loss of connectivity between the SN and the VTA. Both the SN and the VTA contain a high density of dopamine (DA) neurons, and the loss of functional connections with afferent brain regions due to depletion of these DA neurons is associated with Parkinson’s disease onset. The midbrain DA system and its projections to the striatum may be particularly sensitive to TBI. There are numerous preclinical studies reporting damage to the midbrain dopaminergic system and striatum months after moderate to severe TBI (Hicks et al., 1996; Bales et al., 2009; Hutson et al., 2011; Acosta et al., 2015, 2017; Impellizzeri et al., 2016; Liu et al., 2017). The damage is characterized by loss of DA neurons in the SN, increased accumulation of α-synuclein aggregates and putative Lewy bodies, and neuroinflammation marked by activated microglia. The data reported here show rmTBI-induced hypoconnectivity in the SN and VTA, along with altered DWI in the basal ganglia. These data corroborate an expanding literature that head injury is a risk factor for development of Parkinson’s later in life (Jafari et al., 2013; Crane et al., 2016; Taylor et al., 2016; Gardner et al., 2018; Jenkins et al., 2018).

### Resting-State Functional Connectivity: Olfactory System/Cerebellum

One of the more interesting observations from rsFC is the relationship between the olfactory system and the cerebellum. Non-concussed rats showed the olfactory bulb and anterior olfactory n. have close adjacent connections to the orbital and piriform cortices. Six–seven weeks post injury, rats concussed only once showed increased connectivity in the forebrain olfactory system and limbic ctx with hindbrain regions that include the anterior cerebellum (three to five lobules) and deep cerebellar nuclei (lateral and interposed). In contrast, rats exposed to three concussions had reduced connectivity limited only to the olfactory bulb and isolated from the anterior olfactory n.

BOLD imaging in response to odors that involve both systems show brain activation in the olfactory cortex, insula, thalamus, and cerebellum (Pellegrino et al., 2017). Indeed, the cerebellum is consistently activated in human imaging studies that use an odor stimulus (Mainland et al., 2005). While the pathway from the olfactory bulbs to cerebellum has not yet been defined, the circuitry appears to cross over the midline as lesions in the left cerebellum impair odor processing in the contralateral nostril (Mainland et al., 2005). Moreover, data based on changing odor intensity suggest that the intranasal trigeminal system may be responsible for odor-induced activation of the cerebellum (Iannilli et al., 2011; Pellegrino et al., 2017). Disrupted olfaction is commonly found long after initial head trauma in TBI patients (Schofield et al., 2014) and is a highly prevalent, early symptom of Parkinson’s and Alzheimer’s disease (Doty et al., 1988, 1992; Mesholam et al., 1998; Katzenschlager and Lees, 2004).

### Resting-State Functional Connectivity: Cerebellum

The sensitivity of the cerebellum and its efferent connections to the brain through the cerebellar nuclei was examined by seeding the combined dentate, fastigial, and interposed nuclei as well as the posterior cerebellum, and then mapping their connectivity. The cerebellum has reciprocal interactions with much of the brain (Witter and De Zeeuw, 2015). Excitatory outputs from the cerebellar nuclei impact the motor and somatosensory cortices (Allen and Tsukahara, 1974), thalamus (Kalil, 1981; Aumann and Horne, 1996; Aumann et al., 1996), hypothalamus, amygdala, basal ganglia (Hoshi et al., 2005; Bostan et al., 2010; Chen et al., 2014), and hippocampus (Onuki et al., 2015). A single concussion increased connectivity between the cerebellar nuclei and the posterior cerebellum. The primary sensory n. of the trigeminal nerve, vestibular n., parvicellular reticular n., and olivary n. of the underlying medulla oblongata, all of which have reciprocal connections with the cerebellum (Teune et al., 2000; Witter and De Zeeuw, 2015), are part of the enhanced functional circuitry that we observed. The posterior cerebellum showed connectivity in the hindbrain that was similar to the cerebellar nuclei, with additional connections with the limbic ctx, amygdala, and hippocampus. The functional connectivity to these brain regions is consistent with the growing literature on cerebellar involvement in emotion and cognition and its reciprocal connections to these areas (Snider and Maiti, 1976; Heath et al., 1978; Haines et al., 1984; Sacchetti et al., 2002; Schutter and van Honk, 2006; Rogers et al., 2011; Calcagnoli et al., 2015).

The altered connectivity observed after seeding the cerebellar nuclei and posterior cerebellum following three concussions showed a loss of “small worldness,” with reduced connectivity with the cerebellum as well as the underlying brainstem. The motor ctx, basal ganglia, hippocampus, and amygdala showed negative connectivity compared to controls. The sensitivity of these areas to rmTBI may be a risk factor for the cognitive, emotional, and/or motor dysfunction associated with neurodegenerative diseases. For example, similar to our three hit model, Alzheimer’s patients show decreased functional connectivity between the hippocampus and the cerebellum (Allen et al., 2007), and elderly individuals with mild cognitive impairment show reduced connectivity of the hippocampus within a functional network that includes the cerebellum (Bai et al., 2009). In line with our three hit rsFC data, functional connectivity is reduced in Parkinson’s patients between the amygdala and the contralateral cerebellum, and also between the amygdala and the putamen (Hu et al., 2015).

### Limitations and Considerations

This model using momentum exchange for rmTBI and neuroradiology to assess changes in brain structure and function potentially mirrors the human experience and condition. We were unable to identify any changes in cognitive or motor function at 6–7 weeks post injury in one or three hit rats. While it is possible that cognitive and motor deficits would have been revealed with different assays, our assessments showed no overt problems with general health and behavior. However, noninvasive imaging using DWI and rsFC protocols revealed significant alterations in putative gray and white matter architecture and functional connectivity 7–8 weeks post injury, a duration comparable to over 4 years in a human life (11.8 adult rat days = 1 human year) (Sengupta, 2013). In the context of translational neuroscience, this would be sustained injury in humans. The absence of any overt behavioral deficits after an extended period following injury is consistent with mild concussions in humans.

As previously reported, the momentum exchange model for rmTBI produces a constellation of neurological deficits that resolve within a week of head injury (Mychasiuk et al., 2016; Wright et al., 2017). Therefore, it is likely that rats had recovered from these earlier neurological deficits but it cannot be certain. While we tested for cognitive and motor behaviors it would have been of interest had we tested for changes in affective behavior given the reports that head injury in different rodent models of mild TBI increase anxiety (Petraglia et al., 2014; Rowe et al., 2016). Still another, and probably a more relevant behavioral measurement, would have been sleep/waking activity given the loss of functional coupling in the SCN (Castriotta et al., 2007).

These studies were done on adult male rats. Recently, there have been numerous reports addressing sex differences in rmTBI and the vulnerability of adolescence given the increased incidence of head injury in organized sports (Lovell et al., 2003; McCrea et al., 2004; Broshek et al., 2005; Colvin et al., 2009). Wright et al. (2017) clearly showed sex differences in behavior, imaging, and molecular markers in 30–38-day adolescent rats following rmTBI with momentum exchange. RmTBI using control cortical impact on immature 18-day male mice causes changes in white matter FA values and neurochemistry (Fidan et al., 2018). Would the neuroradiological data presented here be different between males and females and dependent upon age of injury?

Recently, there have been many preclinical studies using MRI to interrogate the brain following rmTBI (Wright et al., 2016; Fidan et al., 2018; Meconi et al., 2018; Wortman et al., 2018). DTI is routinely done but the focus is primarily on white matter and only then is predefined areas such as the corpus callosum. The DWI described here with quantitative anisotropy combining a 3D rat MRI atlas with 173 segmented and annotated areas with computational analysis provides an unbiased global interrogation of the brain for subtle changes in gray and white matter microarchitecture (Kulkarni et al., 2015). We are not aware of any preclinical rmTBI studies that use rsFC to follow the long-term consequences of mild head injury. This imaging method is becoming more important in the clinic to diagnose brain function in asymptomatic patients as noted below and focused attention in the clinic on the cerebellum as a biomarker of rmTBI. When our rsFC data are analyzed using the rat MRI atlas, global networks of functional coupling can be reconstructed. Indeed, without this capability the changes in cerebellum and positive correlation with the olfactory system would not have been noted.

It is generally held that the abatement of biopsychosocial deficits is accompanied by a parallel resolution of neuroradiological evidence of brain injury. Indeed, the preclinical images studies cited above show both recovery of behavior and DWI measures shortly after head injury. This was not the case in this study as DWI and rsFC measures in the 1- or 3-hit groups persisted for 7–8 weeks post injury. Similar to the present results, Rajesh et al. (2017) using rsFC reported that neural disruptions and structural insult in mTBI may persist up to 10 years following injury in subjects with normal cognitive function. Hypoconnectivity in the forebrain thought to be responsible for initial cognitive deficits persisted for years after injury and cognitive recovery, suggesting the brain may compensate for disrupted function through reorganization. A time lapse of 7–8 weeks in an adult rat’s life is comparable to 4–5 years in humans (Sengupta, 2013), and thus the continued presence of injury after rmTBI suggests that the hyper- and hypoconnectivity observed in these studies may persist for an extended period.

The site of impact was limited to the rostral cranium at the level of bregma, directly affecting the underlying motor ctx. Although striking this specific site may have produced a unique mechanical force responsible for the observed global changes as described by Mychasiuk et al. (2016), we believe that the neurological effects of TBI are more generalized and agnostic to the site of impact. While concussions can occur on any part of the head, the general neuropathology is reasonably similar among cases. The cerebellum has been recognized as being particularly vulnerable to mTBI (Peskind et al., 2011; Nathan et al., 2015; Meabon et al., 2016; Vergara et al., 2017) and neuroradiological evidence of cerebellar dysfunction has been advanced as a diagnostic biomarker of TBI (Vergara et al., 2017). Our findings of changes in cerebellar connectivity in response to impact to the forebrain support this position. Previously, we addressed whether general markers of dysfunction reliably occur after TBI between subjects and found that concussive injuries to the forebrain or hindbrain of rats result in a similar pattern of neuropathology in the amygdala, hippocampus, and thalamus (Kulkarni et al., 2015).

One of the confounds in preclinical TBI research is the use of anesthesia during head impact as required by many IACUCs. Nonetheless, there are published methods for awake closed head injury and resulting studies with rmTBI in rats reporting behavioral and imaging data that are not dissimilar from that reported with anesthesia (Wright et al., 2016). Anesthesia was used in these studies for both head impact and imaging. The rsFC data were necessarily collected under low dose isoflurane anesthesia to minimize motion artifact and physiological stress (Guilfoyle et al., 2013). Although not optimal, numerous studies comparing anesthetized and conscious states show similar rsFC data (Jonckers et al., 2014; Gorges et al., 2017).

## Conclusion

Recent clinical studies report mild TBI early in life is a significant risk factor for future dementia (Taylor et al., 2016; Gardner et al., 2018; Jenkins et al., 2018; Richardson et al., 2018). The momentum exchange model developed by the National Football League to study player concussions was adapted for use in rats to produce mild concussions without neuroradiological evidence of brain contusions or changes in cognitive or motor behavior. Nonetheless, 7–8 weeks post injury there are significant changes in brain gray matter microarchitecture and function as determined by MRI. The midbrain dopaminergic system and striatum are particularly vulnerable to rmTBI. The sensitivity of the cerebellum to rmTBI corroborates findings in the clinic and may represent a key biomarker in the diagnosis of head injury. Building on the present findings can provide an opportunity to more fully characterize recovery from mild TBI, the efficacy of early intervention strategies to resolve structural and functional alterations, and the risk of dementia later in life associated with mild repetitive TBI.

## Ethics Statement

All methods and procedures described were approved by the Northeastern University IACUC. The Northeastern facility is AAALAC accredited with OLAW Assurance and is registered with the USDA. All housing, care, and use followed the Guide for the Care and Use of Laboratory Animals (8th Addition) and the Animal Welfare Act.

## Author Contributions

CF, PK, NS, and LN: experimental design, resources, and manuscript preparation. PK, XC, SI, TM, and JS: data generation and analysis.

## Conflict of Interest Statement

CF has a financial interest in Animal Imaging Research, the company that makes the RF electronics and holders for animal imaging. NS is a consultant for Azevan Pharmaceuticals, Inc., serves as an officer, and holds equity in the company. The remaining authors declare that the research was conducted in the absence of any commercial or financial relationships that could be construed as a potential conflict of interest.

## Footnotes

1 http://www-sop.inria.fr/asclepios/software/MedINRIA/index.php

2 http://ccni.wpi.edu/

3 http://afni.nimh.nih.gov/afni/

4 http://fsl.fmrib.ox.ac.uk/fsl/

5 https://www.cbica.upenn.edu/sbia/software/dramms/index.html

6 https://www.slicer.org/
