# Supplementary material for: Neuroradiological Changes Following Single or Repetitive Mild TBI
Source: Front Syst Neurosci. 2019 Aug 2;13:34. doi: 10.3389/fnsys.2019.00034 (PMC6688741; doi:10.3389/fnsys.2019.00034)

# **Supplementary Figure 1. Equipment for producing head injury**

Shown below is a picture of the momentum exchange apparatus, the physics for calculating the velocities of impact for different pressures, and the data showing the linear relationship between pressure and velocity. See the supplementary video slowed to 50% speed showing an example of the potential energy following a hit (height of the ball using the equation mass x height x gravity).


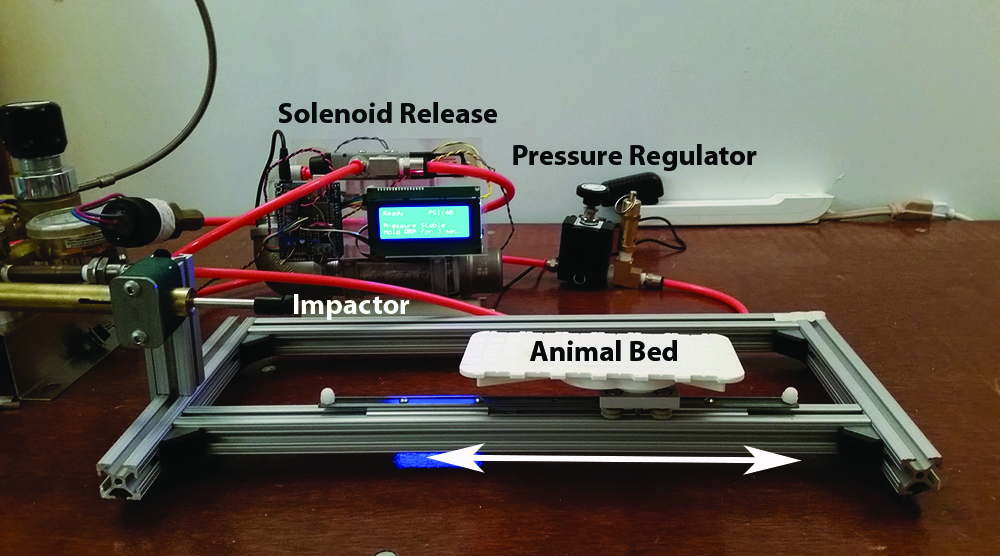


Working with engineers at Animals Imaging Research, LLC (Holden, MA USA) we replicated the pneumatic pressure drive, 50g compactor described by Viano and colleagues and reproduced consistently, the 7.4, 9.3 and 11.2 m/s impact velocities described for mild, medium and severe rat head injury, respectively (Viano, et al. 2009). The impact velocity was regulated by changing the pressure of master cylinder. The relationship between pneumatic pressure and impact velocity was computed using a pendulum and momentum exchange method.

In a simple pendulum with no friction, mechanical energy is conserved. Total mechanical energy is a combination of kinetic energy and gravitational potential energy. As the pendulum swings back and forth, there is a constant exchange between kinetic energy and gravitational potential energy.

When the plunger hits pendulum let us assume that as elastic collision. An elastic collision is a collision in which there is no net loss in kinetic energy in the system as a result of the collision. Both momentum and kinetic energy are conserved quantities in elastic collisions.


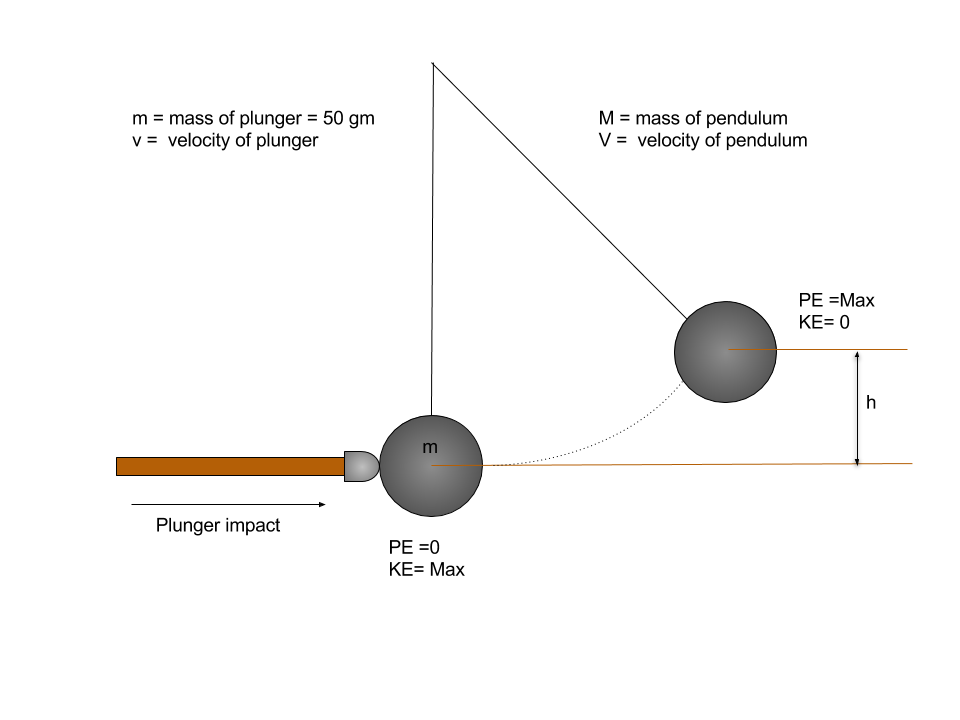


Momentum Exchange Model

Let

$$\mathrm{PE}=Mgh$$

PE = Potential energy of Pendulum at height h

$$\mathrm{KE}=\frac{1}{2}MV^{2}$$

KE = Kinetic energy of pendulum at time of impact

$KE=PE therefore \frac{1}{2}MV^{2}$ = $Mgh$

$$V=\sqrt{2gh}$$

Since this is an elastic system.

$$mv=MV and \therefore v= \frac{M}{m}V$$

$v= \frac{M}{m}\sqrt{2gh}$

Mass of plunger: m = 50 gm

Mass of Pendulum M = 225 gm

| Pressure in PSI | Height of pendulum in meter (h) | Pendulum Velocity in m/s | Plunger Velocity at hit in m/s |
| --- | --- | --- | --- |
| 70 | 0.46 | 3.002665483 | 13.51199467 |
| 60 | 0.37 | 2.692953769 | 12.11829196 |
| 50 | 0.32 | 2.50436135 | 11.26978261 |
| 45 | 0.29 | 2.384114091 | 10.72851341 |
| 40 | 0.25 | 2.213594362 | 9.96117463 |
| 35 | 0.21 | 2.028792744 | 9.12956735 |
| 30 | 0.18 | 1.878297101 | 8.452336955 |
| 25 | 0.145 | 1.685823241 | 7.586204585 |


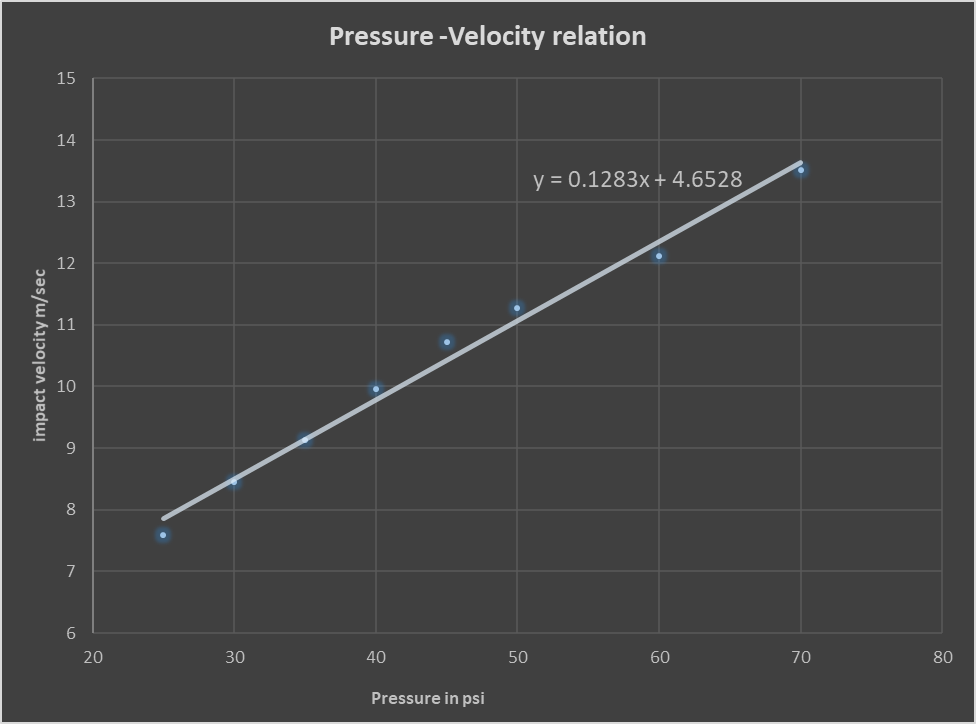

Supplement: Supplementary file 5 [file Data_Sheet_1.docx]
