# Supplementary material for: Neuroradiological Changes Following Single or Repetitive Mild TBI
Source: Front Syst Neurosci. 2019 Aug 2;13:34. doi: 10.3389/fnsys.2019.00034 (PMC6688741; doi:10.3389/fnsys.2019.00034)
Supplement: Supplementary file 6 [file Data_Sheet_2.docx]

**Supplementary Figure 2. Radiograms of Impact Sites**

**
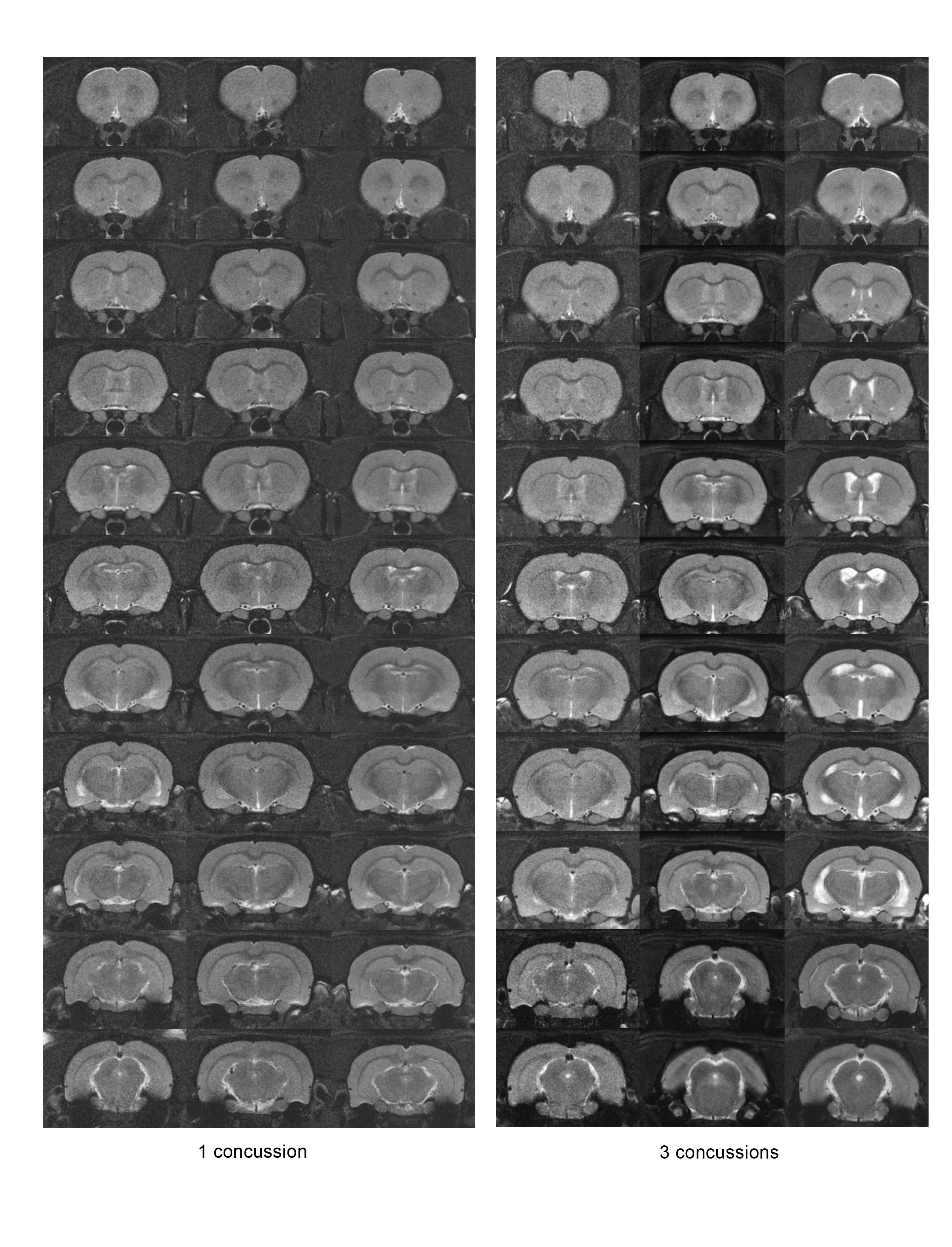
**Shown are radiograms of three rats from each of the one hit and three hit groups. These are 1 mm rostral to caudal, serial, axial sections extending across the midbrain. These are representative of all rats exposed impact and show neuroradiological evidence of contusion.
